# In search of rules behind environmental framing; the case of head pitch

**DOI:** 10.1186/s40462-015-0051-8

**Published:** 2015-09-16

**Authors:** Gwendoline Ixia Wilson, Brad Norman, James Walker, Hannah J. Williams, M. D. Holton, D. Clarke, Rory P. Wilson

**Affiliations:** Department of Geography, College of Science, Swansea University, Singleton Park, Swansea, SA2 8PP Wales UK; Computer Science, College of Science, Swansea University, Singleton Park, Swansea, SA2 8PP Wales UK; ECOCEAN Inc, 102/72 Marine Terrace, Fremantle, WA 6160 Australia; Swansea Lab for Animal Movement, Biosciences, College of Science, Swansea University, Singleton Park, Swansea, SA2 8PP Wales UK; College of Engineering, Swansea University, Singleton Park, Swansea, SA2 8PP Wales UK

**Keywords:** HIPOP, Daily Diary, Head movement, Environmental framing

## Abstract

**Background:**

Whether, and how, animals move requires them to assess their environment to determine the most appropriate action and trajectory, although the precise way the environment is scanned has been little studied. We hypothesized that head attitude, which effectively frames the environment for the eyes, and the way it changes over time, would be modulated by the environment.

**Method:**

To test this, we used a head-mounted device (Human-Interfaced Personal Observation platform - HIPOP) on people moving through three different environments; a botanical garden (‘green’ space), a reef (‘blue’ space), and a featureless corridor, to examine if head movement in the vertical axis differed between environments. Template matching was used to identify and quantify distinct behaviours.

**Conclusions:**

The data on head pitch from all subjects and environments over time showed essentially continuous clear waveforms with varying amplitude and wavelength. There were three stylised behaviours consisting of smooth, regular peaks and troughs in head pitch angle and variable length fixations during which the head pitch remained constant. These three behaviours accounted for *ca*. 40 % of the total time, with irregular head pitch changes accounting for the rest. There were differences in rates of manifestation of behaviour according to environment as well as environmentally different head pitch values of peaks, troughs and fixations. Finally, although there was considerable variation in head pitch angles, the peak and trough values bounded most of the variation in the fixation pitch values. It is suggested that the constant waveforms in head pitch serve to inform people about their environment, providing a scanning mechanism. Particular emphasis to certain sectors is manifest within the peak and trough limits and these appear modulated by the distribution of the points where fixation, interpreted as being due to objects of interest, occurs. This behaviour explains how animals allocate processing resources to the environment and shows promise for movement studies attempting to elucidate which parts of the environment affect movement trajectories.

## Background

Natural selection should act on animals to make them move when they have improved fitness by being elsewhere [1]. This, however, assumes that animals can assess the costs and benefits of remaining stationary, of moving, and of being in the new place, a process that necessitates information [2]. The importance of acquiring information is highlighted by Nathan et al. [3] who, in their conceptual framework integrating drivers of animal movement, point to the critical role of sensory capacities. Indeed, although some information used to inform movement may be in the memory, much is gained, and updated continuously, by sensory systems [2] and it would be surprising, therefore, if there was no clear link between perception of the environment and movement patterns.

Perception of the environment will obviously depend on which sensory systems animals possess, but also, critically, on how these systems are engaged [4], although the details of how much processing capacity animals allocate to various sensory systems cf. [5] are likely to be difficult to determine, particularly in the wild [6]. There is, however, a new approach that might help in this context. Most animals have sensory systems on their anterior ends, presumably to inform them of the conditions ahead. In particular, however, where animals have defined heads, these can also often be moved to deviate from the line of the body and the line of movement to engage in environmental inspection. Such head movement presumably allows animals to assess the conditions outside the direct trajectory taken by the body as part of the continuing overall assessment of the environment. This behaviour has been termed ‘environmental framing’ [7] and its study may help us understand how animals allocate processing time to particular features within the environment and should also help elucidate movement patterns.

Our study here uses a compliant species, humans, to examine the potential that environmental framing has for elucidating the rules animals might have to allocating processing resources to acquiring information. Within studies on humans, much research has concentrated on the development and study of eye-tracking systems [8], which have specific value in helping identification of the particular features of the environment that attract attention [9]. However, eye position is only one facet in a chain of body and head attitudes; the eye movement within the socket amounts to a maximum of 66° [10, 11] while it is actually the orientation of the body and the head that effectively allows for the eyes to have the full coverage of a 360° arc. Seen in this light, the eye can only react to features within the environment that the head and body have ‘framed’. This ‘framing’ behaviour is therefore critical in determining what animals might see. We expect it to depend on what has already been seen, and indeed perceived using other sensory systems, and we also expect some degree of ‘scanning’ to allow animals to gather information from sectors of the environment that are not as compelling, but which are, nonetheless, possibly relevant cf. [12]. An example of this may be an animal feeding but having to scan the environment periodically for predators, even when none have been perceived for some time. The assumption is that the extent of environmental framing, and specifically the arc over which the environment is scanned, will depend on a changing state of the animal’s priorities and the probabilities of the external conditions changing.

To our knowledge, however, only one study has explicitly looked at head movement behaviour within the context of environmental framing. This work, by Wilson et al. [7], presented a methodology for determining head attitude in humans without discussing in any detail patterns that emerged.

Here, we use the system described by Wilson et al. [7], the Human-Interfaced Personal Observation Platform (HIPOP), in an attempt to determine the extent to which environmental framing behaviour, defined by head attitude, is modulated by the environment itself and how much is stylised, perhaps focused towards non-specific information about the environment. For this, we chose to examine head attitude in three groups of people moving within three very different environment types. One group moved along a featureless corridor in a building and so had little external information. One group moved through a ‘green’ space (in a botanical garden), and was operating in a complex environment. A final group moved through ‘blue’ space (snorkelling) through a complex environment, but one that differed radically from the ‘green’ scenario. All participants were navigating through their environment with restricted intent. None were foraging or moving in an environment that was structurally difficult to move through. In addition, both blue and green environment participants were engaged in movement and observation since they were visiting the environments for leisure, while the corridor participants were simply moving with no observation tasks specified. Our aim was to examine one particular aspect of head behaviour, that of head pitch (defining the vertical arc axis of environmental framing) to determine whether there was evidence of rules that could be used to define how the environment is framed by people, and to examine the extent to which this may be changed by the environment type.

## Results

**Fig. 1:**
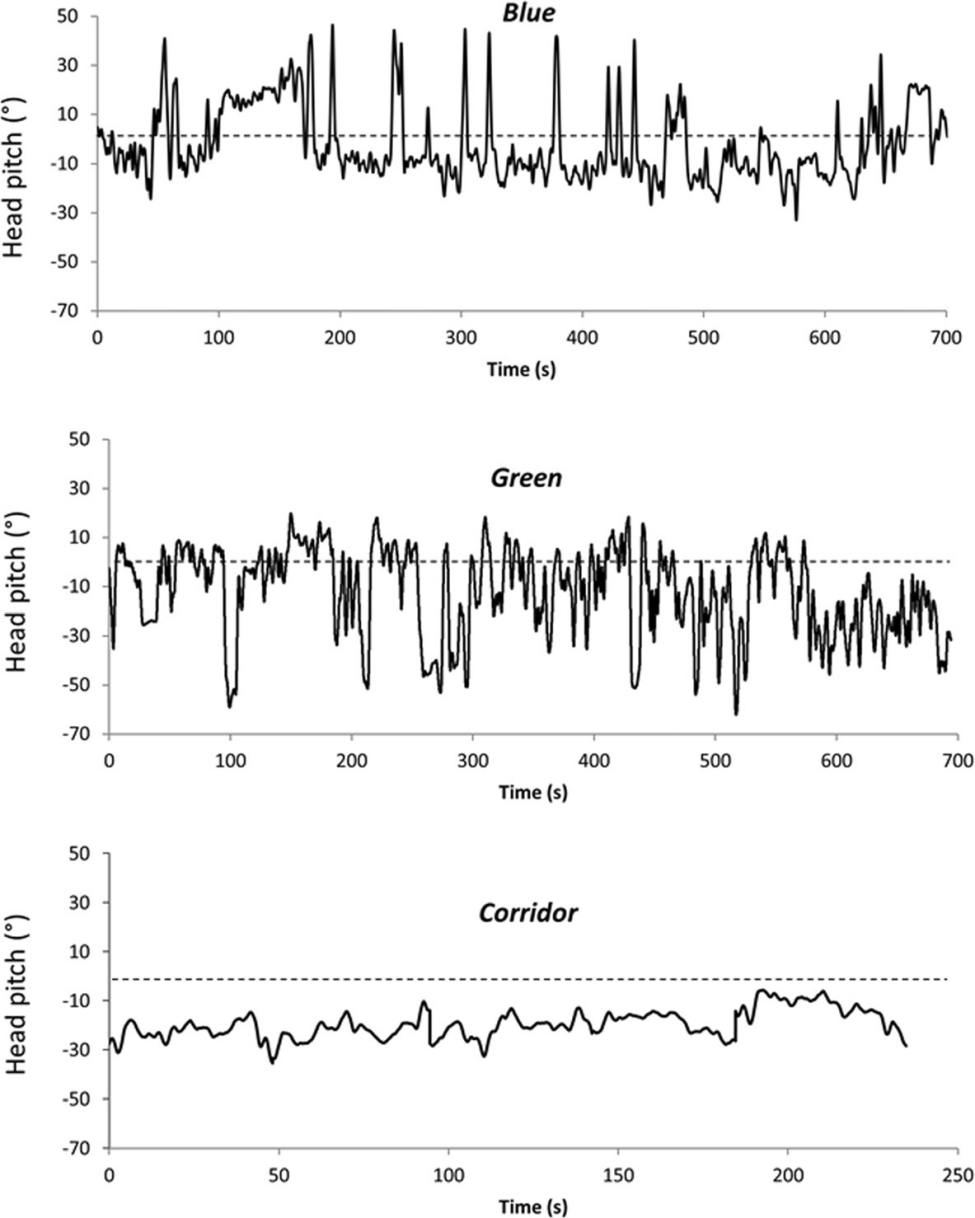
Examples of head pitch over time from participants operating in the three different environments. Note the difference in time scales which explains why the wave frequency appears different between the corridor and other environments but which does not explain why the amplitudes are so constrained in the corridor

**Fig. 2:**
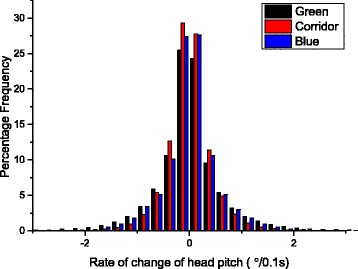
Frequency distribution of the rate of change of head pitch (mean percentage values) for all participants from the three different environments

**Fig. 3:**
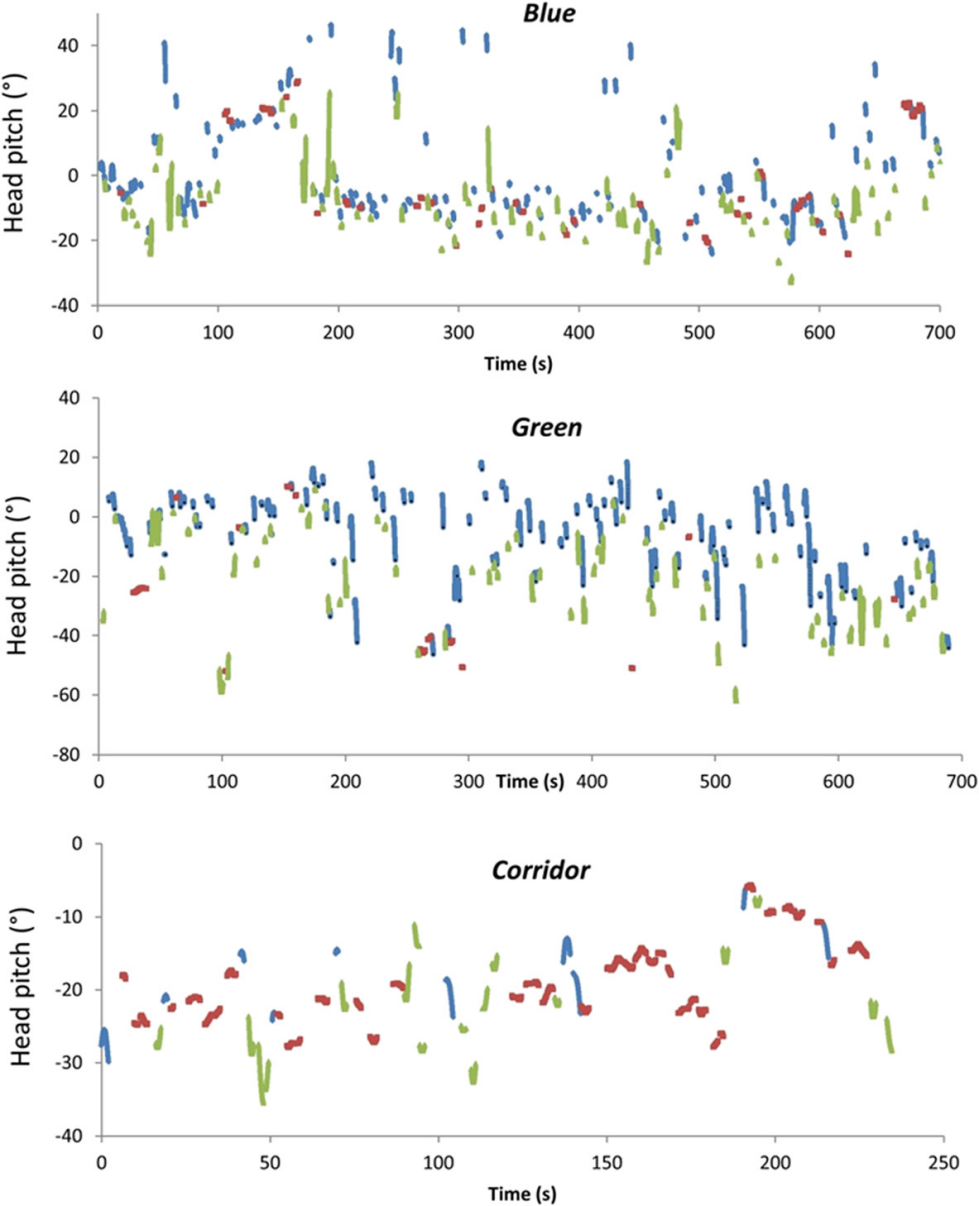
Incidence of stylised peaks [for examples see Fig. 1] (blue symbols), troughs (green symbols) and fixations (red symbols) in head pitch over time in 3 participants operating in 3 different environments (see Fig. 3). Sections of the graphs where there no data are shown correspond to periods where the changes in head pitch did not correspond to the identified stylised behaviour (see text)

Data on head pitch derived from all three environments displayed broadly similar patterns of waveforms with variable amplitude and frequency, with changes in the rate of change of pitch across and within waveforms (Fig. 1). The general similarity across environments was further backed up by very similar overall frequency distributions of the rate of change of head pitch (Fig. 2). The template matching procedure, however, allowed the variability in patterns of head pitch to be quantified, specifically isolating the stylized peaks, troughs and fixations as well as pointing to other areas of the head pitch data where such stylised behaviour did not occur (i.e. where rates of change of head pitch were irregular across time) (Fig. 3). Of note in these data was that behaviours occupied significantly different proportions of time (F = 24.920, df = 2, 111, *P* < 0.001), with more time devoted to troughs (19.93 mean ± 7.03sd %) than peaks (10.87 ± 3.72 %) and fixations (10.09 ± 7.71 %). This relationship held irrespective of the environment where the interacting term was not significant (F = 0.660, df = 4, *p* = 0.621).

**Fig. 4:**
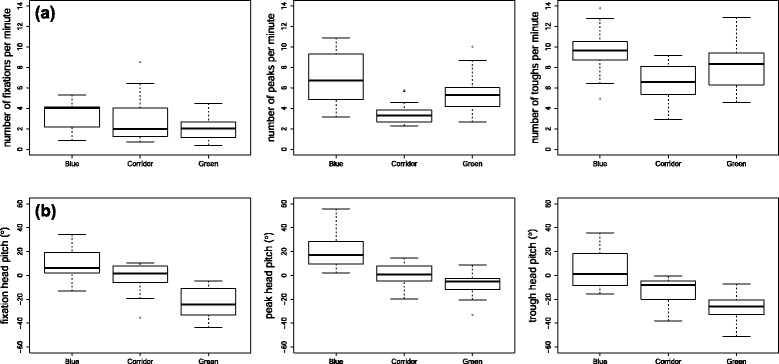
Box whisker plots showing the incidence of various head behaviours as recorded by the HIPOP in different environments (blue, corridor and green). (**a**) shows the rate of manifestation of head behaviours, while (**b**) shows the specific values of head pitch associated with the behaviours corresponding to the graphs immediately above them

The rate of manifestation of behaviours did, however, depend on the environment (F = 2.989, df = 4, *p* = 0.022) in a model with an r^2^ of 0.578. Although the rate of occurrence per minute generally increased from fixations through peaks to troughs, within the corridor environment the rate of occurrence of peaks and troughs was significantly lower than that of both the blue and the green environments (Fig. 4a).

The specific head pitch (in degrees) of the peaks, troughs and fixations, differed by both behaviour (F = 15.009, df = 2, *p* < 0.001) and by environment (F = 50.398, df = 2, *p* < 0.001), although not in interaction, in a model of r^2^ = 0.529 (Fig. 4b).

Finally, a significant relationship was evident between the absolute difference in the mean head pitch between peaks and troughs and the variation (standard deviation) in fixation by individual (t = 2.65, df = 37, *p* = 0.012, *r* = 0.399).

## Discussion

The study of animal behaviour in general has been hugely enhanced by logger technology [13], and specifically the use of accelerometers [14, 15]. Generally though, this approach has dealt with ‘whole body behaviour’ because the tag is attached to the body trunk e.g. [16]. A notable exception to this is work that has examined head movement to allude to the feeding behaviour of animals [17, 18]. To our knowledge, however, within wild animals, no study has used such a system to examine how animals acquire information about their environment. This contrasts to work done on humans where eye-tracking studies are numerous [19]. Here though, the focus is on what features of the environment the eyes actually track [20] rather than examining how the movement of the head frames the environment, as done here. The difference is important because the eyes cannot react to something unless the head has moved so that the element of interest is potentially within the visual field of the eye. Thus, while eye trackers can tell workers what has been the focus of attention, head orientation indicates which sectors of the environment are considered most deserving of attention, and how these change over time.

The starting hypothesis for this work was that head attitude must determine what the animal can perceive while at the same time it was assumed that the environment must modulate head movement because the perceiver should allocate resources (time and sensory attention cf. [21]) to areas and objects of interest. It is well documented, for example, that vigilance in animals can be monitored in a general sense by quantifying the scanning behaviour of the head [22], and that certain conditions will elicit an increased proportion of time allocated to such behaviour [23]. As such, our expectation was that there would be substantive differences in head behaviour between environments. Specifically, we might expect the greatest differences between the corridor and the other two environments because the corridor environment was extremely depauperate in visual (and other) stimuli while the blue and green environments were both rich in stimuli.

In fact, it appears that certain aspects of head behaviour vary little between these environments. In particular, the time allocation to peaks and troughs indicated that reversals in scan direction and scan extents were similar between environments. This is surprising given the variability in the environments and our hypothesis that different environments would elicit different head behaviours. Why might this be? Our data clearly show that waveforms in head pitch are a fundamental feature of the way we inspect our environment, irrespective of that environment and the above data point to two phenomena. These are (i) that there seems to be a fairly standardized rate of change of head pitch (Fig. 2) and (ii) a fairly standard frequency of stylised direction reversals, both of which result in the relative invariance of time allocated to the different behaviours. We suggest that head pitch waveforms are analogous to locomotion, and that there is an optimum rate of movement derived from minimized energy expenditure to execute the movement (analogous to the speed at which minimum cost of transport occurs in locomotion – cf. [24]) and perhaps some optimization of the acquisition of visual information. Clearly, overly rapid head movement increases power demands on the neck muscles but may also result in the environment moving too rapidly over the retina for useful information to be gathered.

In contrast, however, the position of peaks and troughs did vary with environment. Certainly, there was a marked difference in scan arc distribution between ‘blue’ and ‘green’ environments (Fig. 4), and it would be surprising if this were not the case given the fundamentally different practices of snorkelling and walking and the respective attitudes of the head. But there were also differences in scan width (determined by subtracting the mean peak from the mean trough values) between environments, with smaller scan widths in the corridor data (e.g. Fig. 1) than in the other two environments, which implies that the complexity of the environment tends to increase the scan arc. Indeed, it would seem reasonable to suggest that the scan arc extent may be modulated by the distributions of objects of interest, and if the positions of objects of interest are manifest by fixations, then the distribution of objects of interest should correlate to scan width. This is, in fact, exactly what is shown across environments and is even discernible within individuals (cf. Fig. 3). There is, however, a chicken-egg situation here because unless the scan width incorporates an object, it cannot be fixated upon. Clearly, for this reason, our protocol will not give a definitive answer to this but we would suggest that it would be appropriate for animals to base their scan width for environmental framing around objects of interest, but periodically increase the scan arc to incorporate other areas of the environment to ensure that potentially important elements within the environment are given consideration.

Our study is clearly only a very basic starting point. Among other things, we only consider head pitch (when head yaw is presumably also important – cf. [7]), we do not consider eye movement within the eye sockets, participant types were not controlled, and we have effectively ignored about 60 % of all data because we could not categorize it. However, some important trends have emerged. Notably, it would appear that there are some basic rules by which humans, and presumably other animals, might allocate their processing resources to examination of their environment. These rules allow them to concentrate these resources on environmental arcs and objects that have higher priority while still maintaining some coverage of lower priority areas. That animals cannot cover all visual (or other sensory) arcs with equal intensity is clear [21], and is presumably something that predators seek to exploit. But prioritization of relevant arcs for environmental framing is clearly key to enhancing lifetime reproductive success so more studies using HIPOPs on animals should give us a better idea what environmental features animals consider important. If such work is possible in proper environmental context, we might expect it to better our understanding of why animals make decisions to move in the ways they do and help push the movement paradigm to the next level.

## Conclusion

This work exploring the potential of head-mounted devices for determining how animals allocate time and processing resources to examining their environment demonstrates the value of the approach. Although the data only consider head pitch, rather than both pitch and yaw, the study shows that humans continually scan their environment. In particular, people appear to modulate the way the environment is scanned according to the type of environment, specifically concentrating scan arc widths around the distribution of points of interest. Further work using this approach promises to be particularly valuable for assessing how the environment affects the movement trajectories of animals, particularly when the outputs of head-mounted tags can be put into a proper environmental context.

## Methods

### Basic mode of functioning of the HIPOP

The HIPOP is a small head-mounted device that contains orthogonal tri-axial accelerometers (Wilson et al. [7]). Briefly, when these accelerometers are so mounted on the back of the head of a tag-wearer that they measure along the surge, heave and sway axes of the person in question, the smoothed surge axis acceleration values (in *g*) can be sine transformed to provide a good approximation of the pitch angle of the head, following appropriate calibration (see [7] for details).

### Sites

The data for this study was collected on three sites; a corridor on Swansea University campus (51°36'34.623"N, 3° 58'50.266"W) [hereafter the ‘corridor’ group], the National Botanic Garden of Wales (51°50'23.46"N, 4° 9'4.74"W) [hereafter the ‘green’ group], both in Wales, UK, and on boats operating at Ningaloo Reef (22°42'19.08"N, 113° 39'22.679"W) [hereafter the ‘blue’ group], Western Australia.

### Design

This study used a between-subjects design, using 13 participants from Swansea University, 16 at the National Botanic Garden of Wales and 9 participants engaged in the Whale Shark Tours on Ningaloo Reef. Of the total participants 17 were male and 21 female, of ages ranging from 18 to 60. Ethics approval for this study was given from both the Swansea University Biosciences Committee and Murdoch University.

### Apparatus and materials

A HIPOP was head-mounted onto each participant, by attaching it to the back of a cap for the visitors to the National Botanic Garden of Wales (see [7] for details) and those at Swansea University, and to the back of the mask strap for participants snorkelling as part of Whale Shark tours off Ningaloo Reef. Each HIPOP (26 × 27 × 9 mm – mass 10 g) consisted of the elements described for the Daily Diary tag [25]. This logging system contains a tri-axial accelerometer, tri-axial magnetometer and temperature and pressure sensors, which were fitted into plastic casings alongside a rechargeable 300 mAh battery. For the marine deployments the housings they were contained in tied balloons. All devices were set to record at 40 Hz on all channels for the duration of the study.

### Calibration

Precise calibration of the HIPOP for wearer’s head pitch involved each participant standing 1 m away from a metal frame on which there was a marker at the participant’s eye height, as well as one directly 1 m above and 1 m below this. Each subject was instructed to look at each of these spots in turn, giving head pitches of −45°, 0° and 45°. These values were used to correct for marginal errors in the way the HIPOP was mounted on the heads of participants by simple linear regression comparing known values with those predicted from the accelerometers in the tags. This calibration procedure was not possible on the Whale Shark Tour Boats, as the participants took their masks and snorkels off between swims, and recalibration of the devices would not have been possible without disrupting the tour. However, although marginal inaccuracies may have occurred in the absolute values of mask strap-mounted devices, the tags were judged visually to be well placed and we do not expect a systematic inaccuracy.

### Procedure

In each condition, participants were fitted with the HIPOP and the green and blue groups allowed to move freely in their respective environments. The green participants, who were generally in groups of two or three, wherein never more than a single person was tagged, walked of their own volition along a straight, even path that constituted the first part of the botanical garden. HIPOP data were gathered from the blue participants as they snorkelled around Ningaloo Reef in parties of up to 15 members, at times moving over the reef in waters around 5 m deep and at times moving over deeper waters where the seabed was not visible but where pelagic ocean animals could be seen, notably whale sharks *Rhincodon typus.* Participants from the corridor group were instructed simply to walk up and down the length of a featureless, white-walled, corridor four times.

### Analysis

**Fig. 5:**
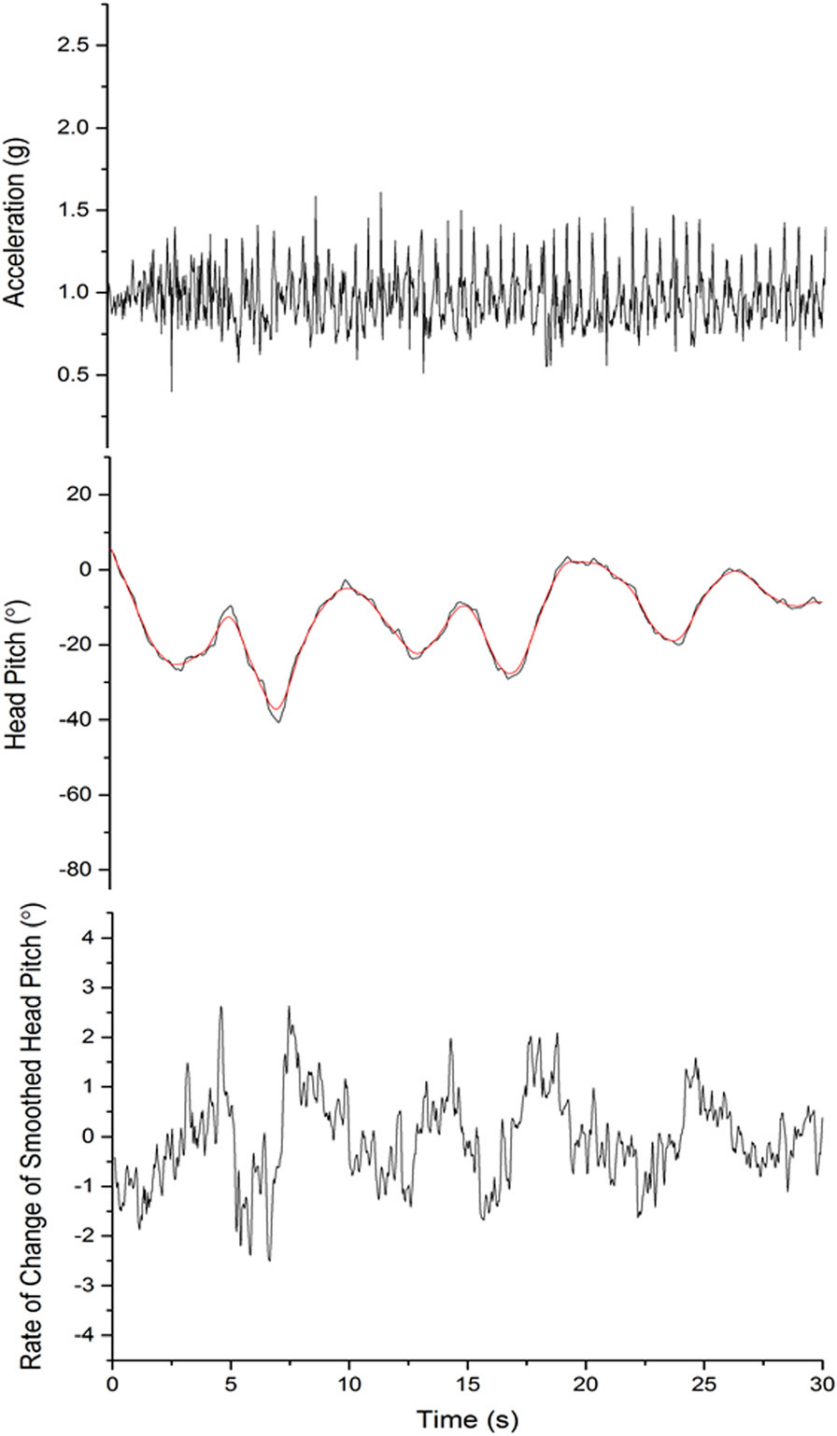
Top panel - Example of the raw acceleration data (taken from the heave axis) from a participant visiting the Botanic Garden to illustrate the obvious waveform due to walking and how (Middle panel) this is manifest in the head pitch (black line) but removed by smoothing (red line). The bottom panel shows the rate of change of the smoothed head pitch for comparison

The participants recorded different amounts of data depending on their activities, with the corridor participants engaged in the task for the least amount of time, followed by the snorkelers and finally by the visitors to the botanic garden. Thus, the analysis was limited to durations of about 120 s per participant for the corridor data and standardized to 700 s for the blue and green datasets. The acceleration data were examined as a function of time in three different formats; as raw surge acceleration, as derived head pitch, and as rate of change of head pitch. This exercise was to determine if there were any obvious repetitive elements within the data set that could be defined as specific environmental framing behaviours, in a manner analogous to defining whole animal behaviours via accelerometry signals [26]. The first obvious pattern was a waveform of about 2 Hz within the data due to changes in body acceleration incurred during walking (Fig. 5). Since this is a by-product of a body, rather than a head, behaviour [27], the data were smoothed over 1 s to eliminate this (Fig. 5).

**Fig. 6:**
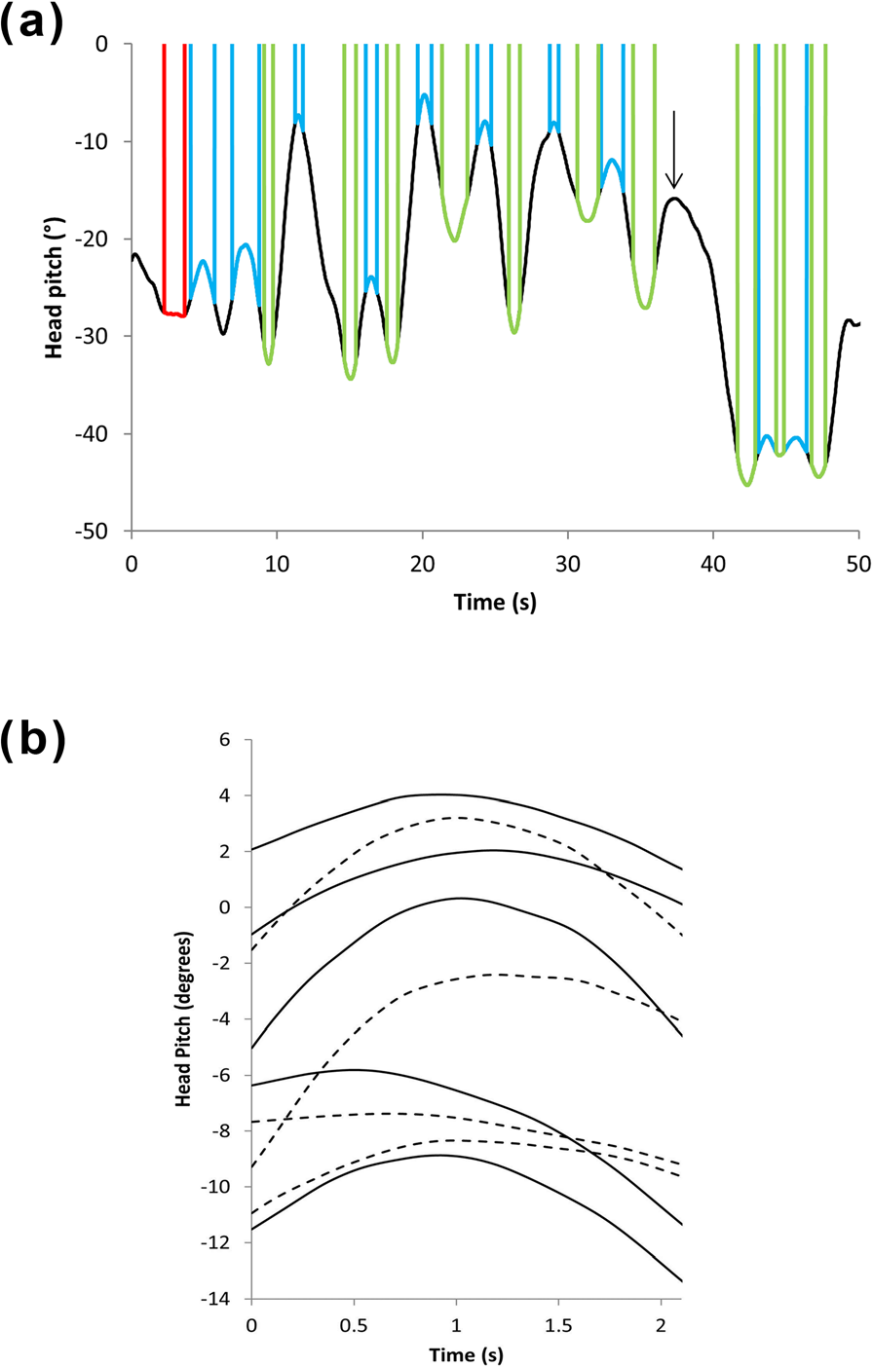
(**a**) Examples of ‘peaks’ in head pitch over time located using FRAMEWORK 4 based on template matching. The variously dashed or continuous lines are only to help visualise separate instances. (**b**) shows peaks highlighted in (**a**) that have been expanded with respect to time to show their form more clearly

Re-inspection of the smoothed data set showed that head pitch varied over time, manifesting itself as waves of varying amplitudes and wavelengths and variable rates of change pf pitch within and between them, interspaced with variable length stable periods. Within this picture, three elements emerged as relatively stylized; (i) smooth wave peaks in head pitch, with rates of change of pitch varying essentially linearly from negative through zero to positive, (ii) smooth wave troughs in head pitch, with rates of change of pitch varying essentially linearly from positive through zero to negative and (iii) head pitch stabilities, where rates of change of head pitch were close to zero for extended periods. Clear examples of these three behaviours, hereafter termed ‘peaks’, ‘troughs’ and ‘fixations’ were used to create templates which were then used to locate these behaviours within the datasets using the template matching process within FRAMEWORK 4 [28]. The program demonstrated how stylized both peaks and troughs were throughout the data (Fig. 6 – this is one where we can plot a few peaks and troughs over each other). Fixations were defined as any head pitch behaviours that had rates of change of head pitch no greater or less than 0.1/s and −0.1/s for a defined period whereby the period had to exceed 1 s in duration.

A series of linear models were performed to quantify the difference in the proportion of the total analysed time that was devoted to each of the behaviours (logit transformed), the display rate per minute and the individual average head pitch (degrees) as predicted by behaviour in interaction with the environment, both as explanatory factors. A Pearson’s correlation was carried out between the absolute difference between means of head pitch at peaks and troughs and the standard deviation of fixations, to identify whether the range in environmental framing is related to the variation in fixation pitch.
